# Chromosome-level genome assembly and annotation of xerophyte secretohalophyte *Reaumuria soongarica*

**DOI:** 10.1038/s41597-024-03644-y

**Published:** 2024-07-22

**Authors:** Miaomiao Song, Wei Gong, Yunyun Tian, Yue Meng, Tingyu Huo, Yanan Liu, Yeming Zhang, Zhenhua Dang

**Affiliations:** 1 Ministry of Education Key Laboratory of Ecology and Resource Use of the Mongolian Plateau & Inner Mongolia Key Laboratory of Grassland Ecology, School of Ecology and Environment, Inner Mongolia University, Hohhot, 010021 Inner Mongolia China; 2 Ministry of Education Key Laboratory of Herbage & Endemic Crop Biotechnology, School of Life Sciences, Inner Mongolia University, Hohhot, 010021 Inner Mongolia China

**Keywords:** Agroecology, Plant ecology

## Abstract

*Reaumuria soongarica* is a xerophytic shrub belonging to the Tamaricaceae family. The species is widely distributed in the deserts of Central Asia and is characterized by its remarkable adaptability to saline and barren desert environments. Using PacBio long-read sequencing and Hi-C technologies, we assembled a chromosome-level genome of *R. soongarica*. The genome assembly has a size of 1.28 Gb with a scaffold N50 of 116.15 Mb, and approximately 1.25 Gb sequences were anchored in 11 pseudo-chromosomes. A completeness assessment of the assembled genome revealed a BUSCO score of 97.5% and an LTR Assembly Index of 12.37. *R. soongarica* genome had approximately 60.07% repeat sequences. In total, 21,791 protein-coding genes were predicted, of which 95.64% were functionally annotated. This high-quality genome will serve as a foundation for studying the genomic evolution and adaptive mechanisms to arid-saline environments in *R. soongarica*, facilitating the exploration and utilization of its unique genetic resources.

## Background & Summary

The drought induced by global warming is intensifying, significantly impacting plant survival and reproduction. This occurrence has led to a cascade of ecological and productivity challenges^1,2^. In arid regions, the coexistence of soil salinity exacerbates the predicament, further complicating agricultural and livelihood practices reliant on plant-based production^2^. In response to stressors like drought and salt, plants have developed sophisticated adaptive strategies at molecular, physiological, and morphological levels. This involves the coordinated interaction among the genome, transcriptome, proteome, and metabolome^3^. Exploring the molecular mechanisms underlying these adaptive responses to adverse conditions has been a longstanding focus of academic inquiry.

So far, significant progress has been achieved in the study of plant stress adaptation^4^. Under these investigations, numerous stress response factors have been identified, and a relatively comprehensive theoretical framework has been integrated. For instance, in drought adaptation, the most representative discoveries include abscisic acid (ABA)-dependent and ABA-independent pathways, DREB2A, and ubiquitination-related mechanisms^5^. In the ABA-dependent pathway, key regulatory genes such as *NFYA5*, *OCP3*, *PLDa1*, *SAL1*, and *MYB96* have been identified for their crucial roles in stomatal regulation, osmotic substance modulation, and lateral root growth under drought stress. Research on salty environment adaptation has highlighted pathways such as the salt overly sensitive (SOS) pathway involved in reconstructing ion homeostasis, reactive oxygen species (ROS) scavenging pathways, and physiologically drought-responsive pathways caused by ion and osmotic substance imbalances^6,7^. Studies on ion transport and homeostasis regulation have revealed that plants primarily utilize Na^+^/H^+^ antiport proteins on the plasma membrane and vacuolar membrane (SOS1, NHX1) to extrude or compartmentalize the influx of Na^+^ into cells, achieve substantial K^+^ uptake through K^+^ channels (e.g., AKT), and reconstruct proton motive force via plasma membrane and vacuolar membrane H^+^-ATPase^8^.

Reports indicate that species thriving in relatively harsh environments have developed unique adaptive strategies through prolonged natural selection and inherent adaptation^9^. It has been proposed that research directions for plant stress adaptation in the “omics” era. On one hand, through multi-omics integrated analysis, further post-genomic research on model plants is being conducted to comprehensively unveil the molecular mechanisms of their stress responses. On the other hand, research is expanding to encompass widely occurring non-model species in nature to obtain additional information on plant stress adaptation, supplementing and refining existing theories^10,11^.

**Fig. 1:**
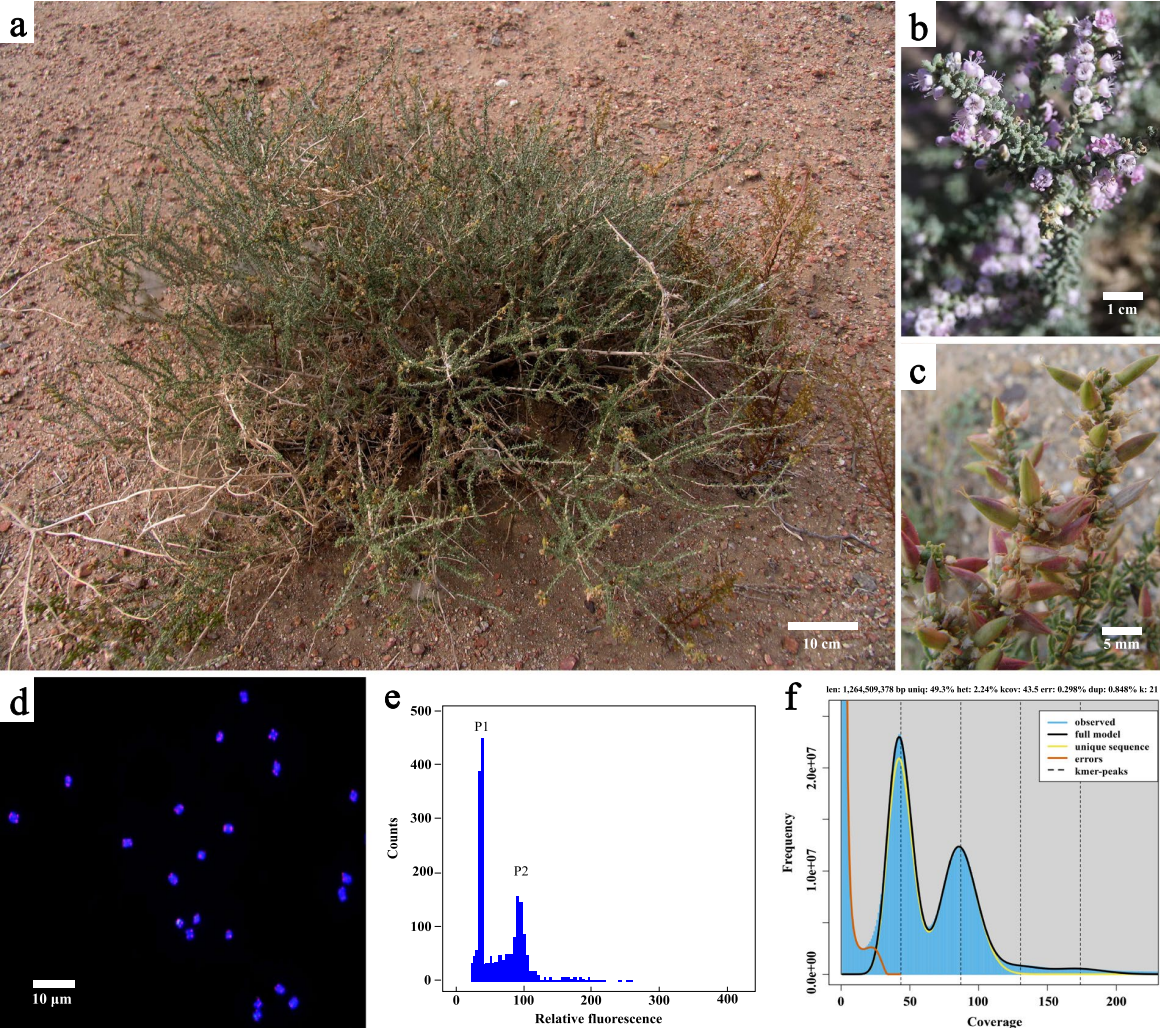
The appearance, genome size, and karyotype analysis of *Reaumuria soongarica*. (**a**), (**b**), and (**c**) represent the whole plant, flower, and seeds of *R. soongarica*, respectively. (**d**) chromosome number and ploidy. (**e**) genome size estimation using flow cytometry. P1 and P2 represent the nuclear DNA contents of *Setaria viridis* and *R. soongarica* samples, respectively. (**f**) K-mer analysis of *R. soongarica* genome. Genome size and heterozygosity rate were estimated using GenomeScope2.

*Reaumuria soongarica* (Fig. 1 a, b, and c) is a shrub belonging to the family Tamaricaceae^12^. This plant exhibits strong resistance to drought, cold, salinity-alkalinity, and barrenness, making it a crucial component of desert ecosystems. The *R. soongarica* community represents the most widespread and extensive zonal community type in arid regions such as Central Asian dunes and deserts^13^. It plays a vital role as an ecological barrier in sustaining and restoring fragile desert ecosystems in Northwest China^14^. *R. soongarica* also serves as a significant forage shrub in desert areas, providing the main source of food for camels throughout the year and for sheep during the winter and spring seasons. Due to its high salt content, livestock can obtain sufficient salt intake from it, stimulating their appetite and promoting weight gain. Additionally, the tender branches and leaves of *R. soongarica* can be used for treating eczema and dermatitis, exhibiting febrifuge and diaphoretic effects.

Here, we assembled a high-quality chromosome-level genome of *R. soongarica* using PacBio HiFi and Hi-C data. The genome has a length of 1.28 Gb, a contig N50 of 116.15 Mb, and a complete BUSCO score of 97.5%. A total of 1.25 Gb of sequences were anchored onto 11 pseudo-chromosomes. Of the genome, 21,791 protein-coding genes, and 60.07% (769.66 Mb) repetitive sequences were identified. This high-quality genome will facilitate the study of adaptive evolution mechanisms in *R. soongarica*, laying the foundation for exploring its unique stress-resistant genetic resources and related molecular mechanisms.

## Methods

### Sample collection

In August 2022, sample collection was conducted in Yihewusu Town (E107°26′02′, N40°11′02′), Ordos City, Inner Mongolia, China. Approximately 20 g of tender leaves were collected from a single plant for genomic sequencing. Following sample collection, the samples were rapidly frozen in liquid nitrogen and transported back to the laboratory for storage in a −80 °C freezer. In the same year, during October, seeds were collected from the same plant in the same sampling site.

### Karyotype analysis

*R. soongarica* seeds were germinated at room temperature. When the roots reached a length of 1.5–2 cm, root tips were treated with nitrous oxide for 2.5 hours. Subsequently, they were immersed in acetic acid for 5 minutes and stored in 75% ethanol. During chromosome preparation, ethanol was removed by rinsing with deionized water. The root apical meristem tissues were dispersed using a mixture of cellulase and pectinase (2:1 ratio). After a 45-minute incubation at 37 °C, the mixture was washed away with deionized water. Once the meristematic tissues were completely air-dried, a 20 µL acetic acid suspension solution was added. After drying the slides, they were examined using an Olympus CX23 microscope (Olympus Corporation, Tokyo, Japan).

For karyotype analysis, well-dispersed intermediate chromosomes were selected. The terminal 21-bp repeat sequence (AG_3_T_3_)_3_ 5′-AGGGTTTAGGGTTTAGGGTTT-3′ was used as a probe^15^. This oligo-probe, synthesized by Sangon Biotech Co., Ltd. (Shanghai, China), was simultaneously tested in a single round of Fluorescence *In Situ* Hybridization (FISH). The hybridization solution, totaling 10 µL, consisted of 1.5 µL of each probe, 8.5 µL of a mixture of 2 × SSC, and 1 × TE, which was dropped onto the chromosomes on a cover glass (24 cm × 50 cm). The slides were then incubated at 37 °C for 2 hours. Using an Olympus BX63 fluorescence microscope combined with a Photometric SenSys Olympus DP70 CCD camera (Olympus Corporation, Tokyo, Japan), the slides were recorded and analyzed. The results revealed a total of 22 detected chromosomes, indicating a diploid plant with a karyotype of 2n = 2x = 22 (Fig. 1d).

### Flow cytometry-based genome size estimation

A quantity of 0.5 g of tender leaves from *R. soongarica* was placed in a culture dish, chopped, and disrupted. Subsequently, 1600 µL of PI solution (staining buffer + PI + RNase storage solution) was added. The mixture was incubated in the dark for 45 minutes and then analyzed using the Sysmex CyFlow^®^ Cube6. The reference species used was *Setaria viridis*, with a genome size of 0.51 Gb^16^. The results estimated the genome size of *R. soongarica* to be approximately 1.26 Gb (Fig. 1e).

### Nucleic acid extraction and quality assessment

For the determination of *R. soongarica* genome size through k-mer analysis and genomic sequencing, genomic DNA was extracted from the leaves using a modified CTAB method^17^. For transcriptome sequencing, *R. soongarica* seeds were sterilized with a 10% sodium hypochlorite solution, rinsed several times with sterile water, and then sown in seedling trays filled with sterile nutrient soil. The cultivation conditions were maintained at 26 °C during the day and 16 °C at night, under a 16-hour light/8-hour dark cycle. The seedlings were watered with 1/2 Hoagland nutrient solution every 3 days. After 3 weeks of cultivation, a healthy plant with intact leaves, stems, and roots was harvested for full-length transcriptome sequencing. Subsequently, seedlings with similar growth were chosen for Na_2_SO_4_ treatment, with three concentration gradients: 0 mmol/L (CK), 200 mmol/L (S200), and 400 mmol/L (S400). After the treatment, tender leaves were collected. Each treatment had three biological replicates, for paired-end transcriptome sequencing. The total RNA of the above-collected samples was extracted using TRIzol^®^ reagent^18^ (Invitrogen, Carlsbad, CA, USA). The isolated DNA and RNA samples underwent quality assessment using NanoDrop-2000 (Thermo Fisher Scientific, Wilmington, DE, USA) and Qubit v3.0 fluorometer (Life Technologies).

### K-mer based genome size assessment

The genome of *R. soongarica* was sequenced using the DNBseq platform. The samples were randomly fragmented using the Covaris ultrasonic high-performance sample processing system, resulting in fragments of approximately 350 bp. Subsequently, DNA fragment end repair was performed, and after passing quality control, the samples were sequenced using high-throughput sequencing. For each qualified library, the raw image data obtained from sequencing were converted into raw sequence data (raw reads) in FASTQ^19^ file format. The SOAPnuke v2.1.0^20^ software was employed for data filtering with the following parameters: (-n 0.02 -l 20 -q 0.4 -i -G 2–polyX 50 -Q 2–seqType 0). A total of 135.09 Gb of clean reads were obtained. The frequency of 21-bp K-mers was calculated using Jellyfish v2.2.6^21^ with default parameters. GenomeScope v2.0^22^ was then utilized to estimate the genome size, heterozygosity, repeat content, and sequencing depth. The results indicated that the estimated genome size of *R. soongarica* is 1.26 Gb, with a heterozygosity rate of 2.24%, and a repeat rate of 50.68% (Fig. 1f).

### HiFi library construction and sequencing

A 20 Kb PacBio library was constructed using the SMRTbell Template Prep Kit-SP v3, following the manufacturer’s instructions (Pacific Biosciences, Menlo Park, CA, USA). Subsequently, Circular Consensus Sequencing (CCS) mode on the PacBio Sequel II platform was employed for sequencing. The raw sequencing data were filtered using smrtlink software v11.0, resulting in a total of 74.62 Gb of clean HiFi data. The filtered CCS data were then converted to a fasta file using samtools v1.18^23^. According to survey-based estimations, the sequencing depth was approximately 58.7×.

### Hi-C library construction, sequencing, and quality assessment

For DNA samples passing quality checks, a sequential process was carried out, including polyformaldehyde cross-linking, MboI enzyme digestion, end repair, biotin labeling, DNA purification, and capture treatment. The Hi-C library was constructed, and paired-end sequencing was performed on the DNBSEQ platform^24^.

The sequencing generated 128.23 Gb of raw data. After filtering out data with adapters and lower quality using Soapnuke v2.1.0, 127.19 Gb of clean data was obtained. The sequencing depth was estimated to be approximately 101×.

### Genome assembly

HiFi reads were assembled into contigs using Hifiasm v0.16.1^25^. Then, purge_dups was performed to remove redundant and erroneous assemblies obtained from the HiFi reads. The contig-level genome was indexed using WBA^26^, and Hi-C data were aligned and merged with the contig-based genome. The generate_site_positions.py program in Juicer v1.5^27^ was used to obtain potential enzyme cut sites in the genome, extracting Hi-C data that uniquely mapped to the genome (Hi-C Contacts) and performing clustering and redundancy removal. Next, 3D-DNA v190716 and Juicer v1.5 were used to scaffold Hi-C reads, constructing a chromosome-level genome.

**Fig. 2:**
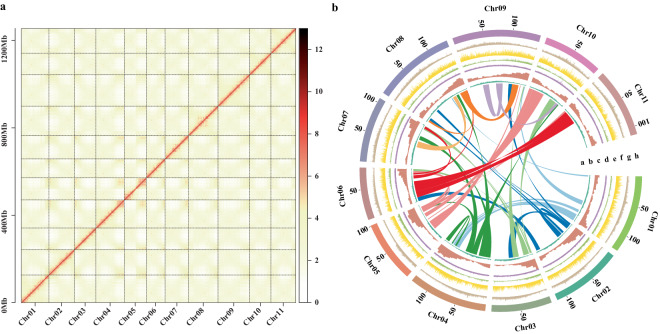
The features of *Reaumuria soongarica* genome. (**a**) Hi-C interaction heatmap of *R. soongarica* genome assembly. (**b**) The landscape of *R. soongarica* genome. The circus plot from the inner to outer represents collinearity blocks (**a**), guanine-cytosine (GC) content (**b**), gene density (**c**), transposable elements (**d**), DNA transposons (**e**), LTR/Copia retrotransposons (**f**), LTR/Gypsy retrotransposons (**g**), and chromosome-scale pseudo-chromosomes (Chr01-Chr11) (**h**), respectively.

The total length of the contig-level genome was 1.28 Gb, approximately matching the K-mer estimate (Table 1). The N50 was 116.15 Mb, and the complete BUSCO score was 97.5%. After Hi-C scaffolding, a total of 1.25 Gb of sequences were anchored onto 11 pseudo-chromosomes, with a scaffolding rate of 97.96% (Fig. 2a and b). The N50 of the chromosome-level genome was 116.15 Mb, and the complete BUSCOs reached 97.8%, indicating a high genome completeness. As shown in Fig. 2a, the Hi-C data signal was strongest along the diagonal, demonstrating effective genome assembly.

**Table 1:** Summary of *Reaumuria soongarica* genome assembly.

Genome assemble	Number/Size
Genome size (Gb)	1.28
Number of scaffolds	327
N50 scaffold length (bp)	116,152,396
N90 scaffold length (bp)	97,711,965
Max length (bp)	144,595,711
Number of contigs	477
N50 contig length (bp)	116,152,396
N90 contig length (bp)	97,711,965
Max length (bp)	144,595,711
GC rate	0.37
LAI	12.37

### Transcriptome sequencing

For assisting genome annotation, both full-length transcriptome sequencing and short-read transcriptome sequencing were performed. Using PacBio’s Single-Molecule Real-Time (SMRT) technology, full-length cDNA was synthesized, PCR amplified, and libraries were constructed. The sequencing was conducted using PacBio’s SMRT technology, resulting in 63.53 Gb of raw data from a *R. soongarica* seedling. A total of 339,291 non-redundant full-length transcripts were obtained, with an N50 of 1,396 base pairs. For short-read RNA-seq, libraries were constructed using a paired-end model and sequenced on the DNBSEQ platform. SOAPnuke v2.1.0 was used for filtering, and HISAT v2.2.1^28^ was employed to align the filtered clean reads to the reference genome sequence. In total, 74.31 Gb of clean data was obtained, with an average output of 8.32 Gb per sample, and the reference genome alignment rate ranged from 94.12% to 95.92%.

### Genome annotation

In this study, a homology-based annotation approach was employed for *R. soongarica* genome annotation. RNA-seq data and full-length transcriptome data were aligned to the genome using HISAT v2.2.1 and Gmap software, respectively. GeMoMa v1.9^29^ was then used to extract intron information from all samples for subsequent auxiliary annotation. Transdecoder was utilized for ORF (Open Reading Frame) prediction on the transcriptome data, resulting in gene annotation based on the transcriptome. Gene structure prediction and information integration were performed using GeMoMa v1.9 for five reference species (*Arabidopsis thaliana*, *Eutrema salsugineum*, *Populus trichocarpa*, *Vitis vinifera*, *Zea mays*). Transcriptome-specific gene information was incorporated, yielding the final gene set. In total, 21,791 genes were predicted in the *R. soongarica* genome (Table 2).

**Table 2:** Summary of gene structure predictions.

	Genes	mRNAs	CDSs
*A. thaliana*	16,020	23,462	139,821
*E. salsugineum*	14,834	17,955	108,034
*P. trichocarpa*	18,048	32,021	193,682
*V. vinifera*	17,717	22,005	124,828
*Z. mays*	13,957	22,678	138,023
Homologs	18,263	18,263	103,672
Transcriptome	3,528	3,528	8,647
Total	21,791	21,791	112,319

Predicted genes were functionally annotated by comparing them to known protein databases using Diamond v0.8.36^30^. The protein databases included SwissProt^31^ (http://www.uniprot.org/), TrEMBL^32^ (http://www.uniprot.org/), KEGG^33^ (http://www.genome.jp/kegg/), InterPro^34^ (https://www.ebi.ac.uk/interpro/), NR^35^, KOG^36^, and GO^37^. The results revealed that 95.64% of genes in the *R. soongarica* genome obtained functional annotation information. Among them, the majority of genes were annotated in the Nr database (20,772, 95.32%), followed by TrEMBL (20,768, 95.31%), and InterPro (18,130, 83.20%) (Table 3).

**Table 3:** Statistics of functional annotation result of *Reaumuria soongarica* genome.

Values	Number	Percentage
Nr	20,772	95.32%
Swissprot	16,176	74.23%
KEGG	16,319	74.89%
KOG	16,389	75.21%
TrEMBL	20,768	95.31%
Interpro	18,130	83.20%
GO	14,154	64.95%
Overall	20,840	95.64%

### Repetitive elements annotation

Two methods were employed for the annotation of transposable elements (TEs), namely the homologous alignment method and *de novo* prediction^38^. The former one was based on the RepBase v21.12^39^ database. It utilizes RepeatMasker v1.332^40^ and RepeatProteinMask v4.0.7^41^ to identify sequences similar to known repeat sequences. *De novo* prediction was based on the assembled *R. soongarica* genome. A *de novo* TE library was constructed using RepeatModeler2^42^ and LTRharvest software^43^. Subsequently, Repeatmasker software was employed for prediction. The results from both methods were then redundantly processed to obtain the annotation of repeat sequences. Tandem repeats were identified by Tandem Repeats Finder v4.09.

**Fig. 3:**
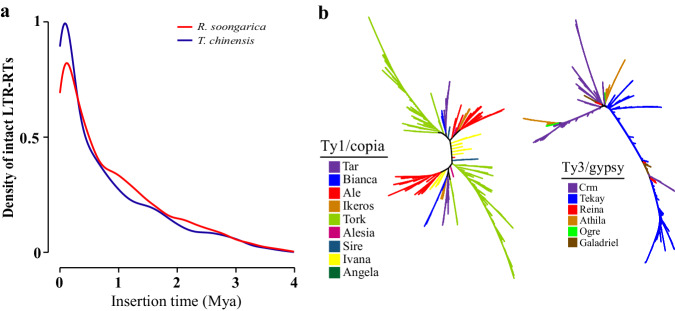
Evolution and classification of LTR retrotransposons (LTR-RTs) in *Reaumuria soongarica* genome. (**a**) LTR-RTs insertion time estimation. (**b**) Clustering analysis of the Ty1/copia and Ty3/gypsy LTR-RTs in *R. soongarica* genome.

In the genome of *R. soongarica*, a total of 769.66 Mb of repeat sequences were identified, constituting 60.07% of the genome. The most abundant repeat elements were the long terminal repeat (LTR) sequences, accounting for 47.47%, followed by DNA transposons at 4.76% and tandem repeats at 3.98% (Table 4). LTR_retriever^44^ was used to identify LTR retrotransposons (LTR-RTs) in the *R. soongarica* genome, and the insertion time of LTR-RTs was estimated. The mutation rate for this estimation was set as 1.52 × 10^−8^, which is twice the mutation rate of Tamaricaceae plants^45–47^. TEsorter v1.4.6^48^ was used for the subfamily classification of LTR-RTs, with Ty1/copia and Ty3/gypsy being the two largest subfamilies in LTRs. Subsequently, iTOL v6^49^ was used to visualize the subfamilies separately in evolutionary trees. The results indicated that LTR-RTs in the *R. soongarica* genome recently burst approximately 0.3 million years ago (Mya) (Fig. 3a). Among them, the most abundant members in the Ty1/copia and Ty3/gypsy families were Tork and Tekay, respectively (Fig. 3b).

**Table 4:** Repeat elements annotation of *Reaumuria soongarica* genome.

Type	Length (bp)	Percentage
DNA	61,026,360	4.76%
LINE	27,001,393	2.11%
SINE	1,319,013	0.10%
LTR	608,163,255	47.47%
Other TE	11,190	0.00%
Unknown TE	21,153,223	1.65%
Tandem repeat	50,984,170	3.98%
Total	769,658,604	60.07%

### Synteny gene identification

**Fig. 4:**
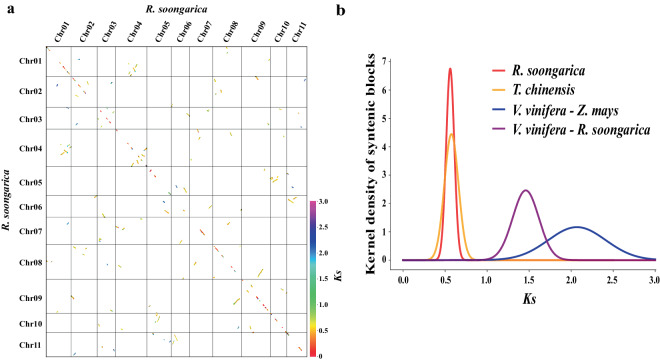
Whole-genome synteny of *R. soongarica* genome. (**a**) Dot plot of synteny blocks in *R. soongarica* genome. (**b**) Distribution of synonymous substitution rate (*Ks*) of syntenic orthologous and paralogous genes among the analyzed genomes.

Synteny genes were identified within and between the genomes of *R. soongarica* and other species (*V. vinifera*, *Z. mays*, and *T. chinensis*). Initially, Python scripts from the Whole-Genome Duplication Integrated analysis (WGDI, v 0.6.2) software^50^ were utilized to generate a modified GFF file for the genome, with the exclusion of alternatively spliced transcripts. Subsequently, Diamond v2.1.6-1 was employed to execute protein-protein BLAST (E-value ≤ 1e^−5^), and the results were formatted in fmt6.blast. Following this, the commands -d, -icl, -ks, -bi, -bk, -c, and -kp of WGDI were successively executed with the default parameters. As a result, 559 synteny blocks were identified in the *R. soongarica* genome, with the gene number ranged from 5 to 103, and the majority of these gene blocks exhibit a 1: 1 relationship in the *R. soongarica* genome (Fig. 4a). The synonymous substitution rate (*Ks*) distribution of the syntenic blocks showed a peak at 0.56 (Fig. 4b).

### Identification of gene families

**Fig. 5:**
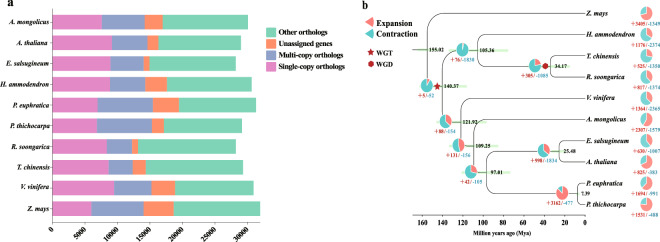
Gene families identification and phylogenetic analysis. (**a**) Orthologous gene groups among analyzed species. (**b**) A phylogenetic tree was constructed based on 793 high-quality single-copy orthogroups from 10 plant species. The numbers of gene-family expansion and contraction on each branch are indicated by red and blue numbers. Numbers on nodes represent the inferred divergence times with 95% confidence intervals.

The genomes of *R. soongarica* and other 9 species (*A. thaliana*, *E. salsugineum*, *P. trichocarpa*, *V. vinifera*, *Z. mays*, *H. ammodendron*, *P. euphratica*, *T. chinensis*, *Ammopiptanthus mongolicus*) were used to cluster paralogous and orthologous groups using Orthofinder v2.5.5^51^. Among 313,500 analyzed genes, 287,806 were successfully assigned to 27,313 (91.8%) orthogroups. For *R. soongarica*, 20,418 genes were identified in 19,517 orthogroups, and 508 were species-specific genes in 160 orthogroups (Fig. 5a). Across the ten genomes, 793 single-copy orthologous were identified and aligned using MAFFT v7.505^52^, trimmed using trimAl v1.4.rev15^53^. The optimal model was used with IQ-TREE v1.6.11^54^ to analyze the tree and infer the divergence dates. Trees of the 10 species were constructed with the ML model using RAxML with 1000 bootstrap replicates v8.2.12^55^, and the best-obtained model was GTR + F + R4. The divergence time was calculated using the MCMCtree program in PAML v4.9 h^56^. The divergence time between *Z. mays* vs *V. vinifera* (142.1–163.5 Mya) and *E. salsugineum* vs *A. thaliana* (19.7–34.2 Mya) acquired from TimeTree (http://www.timetree.org/) was used as the calibration points. The phylogenetic analyses verified that *R. soongarica* was a sister to *T. chinensis*, with strong bootstrap support (>50%), and the two species diverged nearly 34.17 Mya (95% HPD = 30.04, 113.1) (Fig. 5b).

CAFE v5.0^57^ was performed to identify expansions and contractions of gene families in *R. soongarica* genome. 218 and 196 gene families were found to have significantly expanded and contracted, comprising 1,303 and 207 genes, respectively.

### Identification of salt-stress response gene

**Fig. 6:**
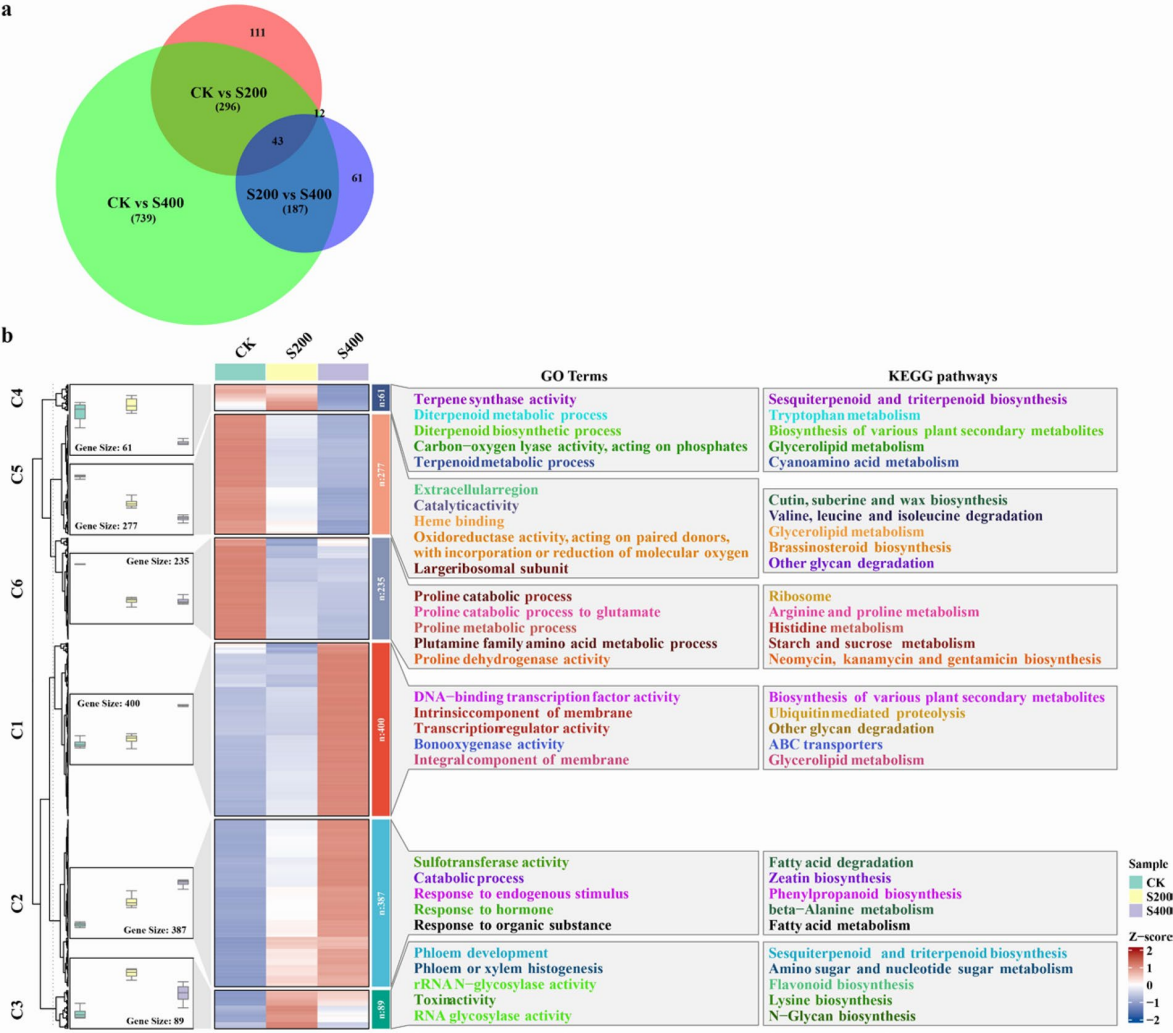
Differentially expressed genes (DEGs) identified from the salt-stressed *R. soongarica* tranccriptomes. (**a**) Venn diagram of DEGs under different salt concentrations. (**b**) Expression patterns, clustering, and functional enrichment analysis of DEGs under varying salt concentrations. C1 to C6 represent DEG clusters identified by clustering analysis.

Differential expression genes (DEGs) were identified using DESeq2^58^, with the criteria of |Log_2_FC| > 1 and false discovery rate (FDR) < 0.05 for filtering. A total of 1,449 DEGs were identified through pairwise comparisons (Fig. 6a). Cluster analysis demonstrated distinct expression patterns of these DEGs under different salt concentrations. Functional enrichment analysis showed that these DEGs were enriched in various GO terms and KEGG pathways that may relate to salt-stress response in *R. soongarica* (Fig. 6b).

## Data Records

All sequencing data described in the study have been deposited in the NCBI database. PacBio HiFi long reads, Hi-C reads, and full-length transcriptome Iso-Seq reads were deposited in the Sequence Read Archive (SRA) under accessions SRR27540885^59^, SRR27540886^60^, and SRR27540882^61^, respectively. The RNA-seq data of salt-stressed samples were deposited in the SRA under accessions SRR27540875-SRR27540881^62–68^, SRR27540883^69^, and SRR27540884^70^, and the genome survey data with SRA accession number SRR28495917^71^. The genome assembly and annotation of *R. soongarica* has been deposited on the Figshare platform^72^, and GenBank with accession number JBEBFM000000000 (2024)^73^, respectively.

## Technical Validation

**Fig. 7:**
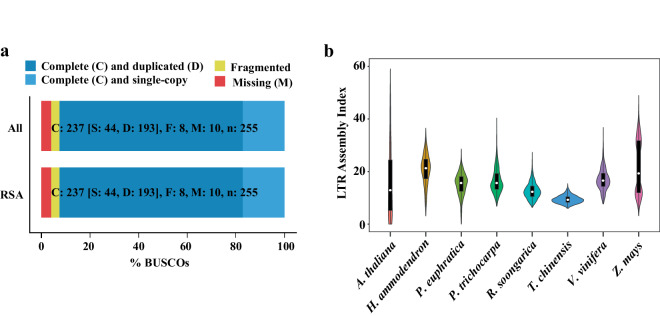
Quality assessment of the assembled *Reaumuria soongarica* genome. (**a**) BUSCO analysis of the full-length transcriptome. (**b**) LTR assembly index for *R. soongarica* and the referenced genomes.

For the genome assembly, we assessed the quality using BUSCO v5.2.2, embryophyta_odb10). The assembly achieved completeness of 97.5% at the contig level and 97.8% at the chromosome level, indicating a highly complete genome (Table 5). For the full-length transcriptome sequencing data, we employed the single-copy ortholog database BUSCO to assess the quality of the assembled transcripts (Fig. 7a). Additionally, 97.96% of the Hi-C data were successfully anchored to the 11 pseudo-chromosomes, confirming the accuracy of the chromosome assembly (Fig. 2a). The organization of interaction contacts within and around the chromosome region was observed through the Hi-C heatmap, further supporting the quality of the chromosome assembly. We also calculated the LTR Assembly Index (LAI) using LTR_retriever v2.9.0, obtaining a value of 12.37, indicative of a genome of reference quality (Fig. 7b, Table 1).

**Table 5:** Evaluation of *Reaumuria soongarica* genome assembly.

Type	Contig level	Chromosome level
Complete BUSCOs (C)	97.5%	97.8%
Complete and single-copy BUSCOs (S)	93.9%	94.1%
Complete and duplicated BUSCOs (D)	3.6%	3.7%
Fragmented BUSCOs (F)	0.7%	0.4%
Missing BUSCOs (M)	1.8%	1.8%

## Data Availability

All data processing commands and pipelines were carried out in accordance with the instructions and guidelines provided by the relevant bioinformatic sofware. There were no custom scripts or code utilized in this study.
