# Publisher Correction: Chromosome-level genome assembly and annotation of xerophyte secretohalophyte *Reaumuria soongarica*

**DOI:** 10.1038/s41597-024-03935-4

**Published:** 2024-10-18

**Authors:** Miaomiao Song, Wei Gong, Yunyun Tian, Yue Meng, Tingyu Huo, Yanan Liu, Yeming Zhang, Zhenhua Dang

**Affiliations:** 1 Ministry of Education Key Laboratory of Ecology and Resource Use of the Mongolian Plateau & Inner Mongolia Key Laboratory of Grassland Ecology, School of Ecology and Environment, Inner Mongolia University, Hohhot, 010021 Inner Mongolia China; 2 Ministry of Education Key Laboratory of Herbage & Endemic Crop Biotechnology, School of Life Sciences, Inner Mongolia University, Hohhot, 010021 Inner Mongolia China

**Keywords:** Agroecology, Plant ecology

Correction to: *Scientific Data* 10.1038/s41597-024-03644-y, published online 22 July 2024

In this article, the scale bar in figure 1b is missing. Second, the positing of the star in figure 5 is incorrect. The original article has been corrected.

